# Treatment-Related Dysgeusia in Oral and Oropharyngeal Cancer: A Comprehensive Review

**DOI:** 10.3390/nu13103325

**Published:** 2021-09-23

**Authors:** Lucrezia Togni, Marco Mascitti, Arianna Vignigni, Sonila Alia, Davide Sartini, Alberta Barlattani, Monica Emanuelli, Andrea Santarelli

**Affiliations:** 1 Department of Clinical Specialistic and Dental Sciences, Marche Polytechnic University, 60126 Ancona, Italy; togni.lucrezia@gmail.com (L.T.); m.mascitti@pm.univpm.it (M.M.); s.alia@pm.univpm.it (S.A.); d.sartini@staff.univpm.it (D.S.); m.emanuelli@univpm.it (M.E.); andrea.santarelli@staff.univpm.it (A.S.); 2 Department of Clinical Sciences and Translational Medicine, Tor Vergata University, 00133 Rome, Italy; barlattanialberta@gmail.com; 3 Dentistry Clinic: National Institute of Health and Science of Aging, IRCCS INRCA, 60124 Ancona, Italy

**Keywords:** dysgeusia, taste disorders, oral squamous cell carcinoma, oropharyngeal squamous cell carcinoma, chemotherapy, radiation therapy, surgical oncology

## Abstract

Oral cancer is the most common tumor of the head and neck region. Its management is based on surgical and systemic therapies. Taste disorders represent the most common side effect of these treatments; indeed, dysgeusia is noted by 70% of oral cancer patients. Despite survival remaining the primary endpoint of cancer patients, taste impairments can cause psychological distress. This comprehensive review describes the last decade’s knowledge from the literature regarding taste alterations in patients with oral and oropharyngeal squamous cell carcinoma. A total of 26 articles in English, including prospective, cross-sectional, and case–control studies, and clinical trials were evaluated. Literature analysis shows that anti-cancer treatments can destroy taste cells, decrease and alter their receptors, and interrupt nerve transmission. Furthermore, the tumour itself can destroy the oral mucosal lining, which encloses the taste buds. Dysgeusia typically occurs in 3–4 weeks of treatments, and usually taste sensation is recovered within 3–12 months. However, some patients exhibit incomplete or no recovery, even several years later. Thus, dysgeusia can become a chronic issue and negatively influence patients’ quality of life, worsening their dysphagia and their nutritional status. Physicians should be focused on preventing oncological treatment-related symptoms, offering the most suitable personalized support during therapy.

## 1. Introduction

Oral squamous cell carcinoma (OSCC) is the most common tumour of the head and neck (H&N) region and the sixth most common worldwide neoplasm, accounting for more than 90% of oral malignancies [1]. Over 400,000 new cases are diagnosed annually, and the mortality rate remains among the highest, and has been stable for over 20 years [2]. The short-term survival rate is less than 50% and the available treatment strategies do not show favorable medium to long term efficacy [3]. The incidence of oropharyngeal squamous cell carcinoma (OPSCC) has significantly increased in recent years due to the carcinogenic effects of human papillomavirus (HPV) [4]. The increase of its incidence, combined with the younger age of onset and the improved prognosis associated with HPV infection, highlights the need to optimize the survival outcomes in OPSCC patients. The oncological treatment of OSCC is based on surgical techniques and adjuvant and/or neoadjuvant medical therapies, based on the pathological stage and adverse risk features [5]. Their side effects include a wide range of acute and late toxicities, such as orofacial pain, oral fibrosis and/or mucositis, dysphagia, dysgeusia, xerostomia, salivary impairments, osteonecrosis of the jaws, nausea, fatigue, and dermatitis. These alterations can become chronic issues and negatively influenced patients’ quality of life (QOL) [6,7,8,9,10,11,12,13]. Taste disorders include hypogeusia (reduced taste sensation), ageusia (loss of taste sensation), dysgeusia (altered taste sensation), phantogeusia (taste alteration without external stimulus) and parageusia (distortion to a specific stimulus). Despite the various terms, the most used, as the general definition for any alteration of normal taste, is “dysgeusia” [14]. Taste perception is mediated by taste receptor cells, bundled in clusters called taste buds. Each taste bud contains 40–120 cells, classified into taste receptor cells, support cells, and precursor cells. Taste buds are mainly located on the tongue dorsum, but can also be found on the buccal mucosa, floor of the mouth, oropharynx, epiglottis, pharynx, larynx, and in the upper third of esophagus [6,7,13,15,16]. Some taste cells were estimated to have a half-life of 8–12 days, while others reached a 24-day half-life [15,16]. Taste signals are transmitted by several nerves: the chorda tympani (branch of facial nerve), the glossopharyngeal, the upper laryngeal (branch of vagus nerve), and the lingual (branch of trigeminus nerve). The proprioceptive sensitivity carries the touch, thermic and positional sensations, as well as the gustatory impressions that enable us to appreciate food taste and quality. Although the oral phase of swallowing is voluntary, it depends on the integration of mechanical (through contact of foods) and chemical (through smell and taste) stimuli, preparing the entire gastrointestinal system to receive the food [13]. Therefore, taste impairment significantly reduces QOL in cancer patients, worsening their dysphagia and their nutritional status, leading to failed healing and postoperative complications. The exact mechanism underlying the taste dysfunction in cancer patients is yet unknown. The literature studies include heterogeneous cancer populations with a wide range of symptoms, difficult to correlate with specific biologic markers [17]. Although survival remains the primary endpoint, taste alteration represents the commonest side effect of cancer treatments; however, this clinical manifestation is still poorly studied. Therefore, the aim of this comprehensive review is to analyze the literature published in the last decade regarding taste impairments in patients affected by OSCC and OPSCC. This study will especially focus on the impact of oncological treatments in taste alterations, overviewing the etiological hypotheses and the available therapeutic strategies.

## 2. Treatment-Related Dysgeusia in Oral and Oropharyngeal Cancer Patients

### 2.1. Multimodality Therapy

Dysgeusia may begin as mucosal damage due to cytotoxicity and neurotoxicity from radiation therapy and systemic medications [18]. These treatments can destroy high turnover taste cells, decrease their receptors, alter their cell structure, induce receptor surface changes, and/or interrupt the nerve transmission. The loss of taste progenitor cells may result in a reduced recovery rate of damaged taste buds. Thus, a chronic taste alteration may reflect decreased turnover of receptor cells, lack of connectivity between receptor cells and neurons, and neuronal damage [18]. According to Galitis et al., taste disorders are one of the most important symptoms reported by OSCC patients who have undergone chemoradiotherapy (CMRT) [19]. It is estimated that dysgeusia is experienced by approximately 76% of patients who undergo H&N-CMRT. Taste changes seem to significantly worsen between the 4th and the 8th week of CMRT, peaking during the 8th week of treatment. Furthermore, taste symptoms have been associated with treatment modality (RT vs. CMRT), cumulative radiation dose, and smoking status [20,21]. Indeed, smokers who have undergone IMRT and/or CMT reported a significantly higher rate and a worse severity of taste loss compared to non-smokers (dysgeusia rate: 100% smokers vs. 36% non-smokers; severe dysgeusia: 100% smokers vs. 9% non-smokers) [18]. Smoking, altering the gustatory function, affects taste differentiation. This suggests that the smokers’ gustatory recognition is lower than non-smokers, making it necessary to increase the concentration of the proposed stimulus for proper recognition of taste [13]. The toxicity of multimodal treatments significantly affects the gustatory acuity of OSCC and OPSCC patients. The type of treatment, cumulative dose and patient status strongly influence the subjective symptoms. The literature study, concerning dysgeusia induced by multimodality therapy, is displayed in Table 1.

### 2.2. Chemotherapy

Chemotherapy (CMT) significantly affects taste; indeed, taste impairments are experienced by 46–77% of OSCC patients who have undergone CMT [16], which defines dysgeusia as the worst side effect of treatment [37]. CMT may have cytotoxic effects via systemic distribution and direct effects via secretion in saliva and gingival crevice fluid. CMT alterations could be caused by the impairment of taste cells’ proliferation and repair, and by its pharmacological cytotoxicity [18]. CMT may also affect neuronal activities. Abnormal sensitization of the chorda tympani nerve can result in specific taste sensations without stimulating the taste receptors or requiring the presence of the corresponding flavor molecules [18]. Altered taste has been found to occur consistently within 3–5 days of starting CMT, and it usually returns within 3 weeks, although there have been reports of persistence of symptoms for more than 6 months [6,15]. Dysgeusia represents an early symptom that drastically worsens the QOL of patients undergoing CMT, by direct and indirect effects on cell proliferation and neuronal activity. Table 1 summarizes the scientific studies concerning CMT-induced taste disorders in OSCC and OPSCC patients.

### 2.3. Radiation Therapy

Accumulating evidence shows that the impairment of basal and differentiated taste cells is the main cause of radiotherapy-induced taste dysfunction [18]. Radiation therapy (RT) may alter the structure of taste pores, leading to a disrupted delivery of flavor molecules to receptor cells, or a thinning of the papilla epithelium. The hypotheses to explain irradiation-induced taste impairment include inflammation of afferent nerves that supply taste buds, direct damage to differentiated taste cells, and ablation of proliferating progenitors, preventing the renewal of taste cells [7]. Especially, the suppression of the basal cells seems to decrease the type II taste cells, considered to be stimulus receptor cells [29]. Despite the fact that in the pre-therapy phases, the patients rank potential dysgeusia as least important toxicity, at the end of therapy and during the follow-up period the reduction of taste becomes a major issue affecting the patients’ QOL [7]. Long-term objective taste impairments have been reported by 70% of irradiated patients affected by OSCC [7,18], while dysgeusia has been reported by 40% and 88% of patients before and after RT, respectively. Moreover, a 3-fold increase in symptoms has been demonstrated during treatment [19]. The radiation doses to healthy organs such as the tongue or parotid glands are roughly estimated; moreover, the tolerance doses vary widely, ranging from 27 to 65 Gray. Nevertheless, almost all patients experience a significant loss of taste acuity at a dose of 60 Gray. Dysgeusia typically occurs within 3–4 weeks of treatment and some studies have shown complete recovery within 3–12 months, depending on the volume of the irradiated tissues [6,12,15,16]. Especially moderate taste alterations start during the 2nd week of RT, involving 40% of patients, while severe dysgeusia is experienced from the 3rd week onward, reaching the peak of rate and severity of symptoms in the 5th week [11]. Unfortunately, some patients exhibit incomplete or no recovery even several years later [6,12,15,16]. Indeed, significant worsening of taste function both at short (within 1 year) and long term (up to 11 years) follow-up was shown in survival and non-survival patients with OSCC who have undergone adjuvant RT, compared to the baseline [6,17,28]. Similar results were found in early-stage OTSCC [32] and OSCC [27], 6–12 months after adjuvant RT. This deterioration seems to be due to treatment sequelae rather than disease consequences, although ageing could compromise long-term results. New RT approaches, such as conformal and modulated RT, seem to significantly reduce the late toxicity rather than conventional RT [16]. Recently, Vempati et al. showed a high prevalence (90%) of acute (<90 days) and tardive (>90 days) dysgeusia after stereotactic radiosurgery. The mild alterations accounted for 56% and 65% of acute and tardive toxicities, respectively, whereas moderate dysgeusia was reported in 32% and 24% of acute and late toxicities, respectively. Despite a gradual return to baseline scores, moderate dysgeusia was experienced by 7% of patients at 24 months post-treatment [23]. In recent years, the use of intensity-modulated RT (IMRT) has allowed for effectively reducing radiation doses. Important organs, such as the major and minor salivary glands, oral mucosa, and maxillofacial muscles, can be partly spared from high doses of radiation, reducing toxicities without affecting tumor control rates. Especially, in OSCC-patients, helical IMRT demonstrated better radiation protection compared to traditional IMRT. However, according to Moroney et al., all advanced OPSCC patients undergoing helical IMRT and concurrent CMT reported dysgeusia during the last week of therapy and in the following two weeks. In particular, symptomatic patients progressively increased from the baseline (40%) to the end of therapy (100%), and a sharp increase in incidence was seen from the 3rd to 4th week of treatment due to the second cycle of high-dose cisplatin. Additionally, the severity of dysgeusia progressively increased. Finishing IMRT, almost all (98.4%) patients experienced a moderate dysgeusia. Taste symptoms and their severity tend to decrease during the post-operative period; however, more than 80% of patients experienced taste disfunction, even 12 weeks after treatment. Moreover, 3 months later, moderate and mild dysgeusia continued to be observed in 26.4% and 54.7% of patients, respectively [25]. Sapir et al. found a significant correlation between severe dysgeusia and mean oral cavity IMRT doses. The authors reported a substantial worsening of incidence (from 20% to 50%) and severity (from mild to severe) of dysgeusia one month after the start of IMRT. Three months later, there was a gradual improvement of symptoms, reaching 35% of cases with severe dysgeusia induced by a mean oral cavity radiation dose equal to 53–57 Gray. Over time, the risk of severe RT-related dysgeusia declined regardless of the dose employed; however, 12 months later, about 20% of patients still experienced severe dysgeusia [7]. Proton beam therapy (PBT) has also been proved to minimize the dose to the surrounding normal tissues. According to Hayashi et al., no patients experienced severe dysgeusia; however, moderate taste disorders were experienced from 60% of patients treated by PBT [30]. Another highly effective method to reduce radiation-induced toxicity involves the use of customized oral stents. Cerrobend is the most common alloy used for these stents; however, Yanchen et al. failed to demonstrate any significant advantage of Cerrobend stents in minimizing the taste alterations in OSCC irradiated patients [9].

The impairment of type II taste cells seems to be the main cause of RT-induced taste dysfunction. The prevalence and severity of dysgeusia culminate during the last weeks of RT and most patients complain of incomplete or no taste recovery, even several years later. Symptoms and tolerance doses are widely variable relating to the volume of irradiated tissues. Novel RT approaches significantly reduce the radiation doses and the late toxicities; however, they did not significantly improve taste disorders. The literature study of RT-induced dysgeusia in OSCC and OPSCC patients is reported in Table 1.

### 2.4. Surgical Treatment

Surgical resection, with or without adjuvant therapies, is the gold standard treatment for curative management of OSCC. The extent of surgical resection and the reconstruction modalities can affect the sensation of taste [6,15,26]. The tumour itself can also potentially destroy the oral mucosal lining, which encloses the taste buds. The tumour may also compromise the food bolus preparation and prevent the aroma from passing from the pharynx up to the nasal olfactory epithelium [26]. According to Tomita et al., 50% of OTSCC patients who have undergone partial or subtotal glossectomy complain of taste disorders and their taste threshold results are significantly higher than the non-oncological controls. The capacity of patients to recover taste after surgery seems to depend on the extent of tissue excision. Especially, the severity of symptoms depends on surgical preservation of the oropharynx. The conservation of more than 50% of the tongue base is associated with a better taste sensation and a lower detection threshold, because of a lower amount of damage to the glossopharyngeal nerve [6]. The introduction of flap reconstruction in cervical-cephalic oncologic surgery allowed for a better surgical radicality. One of the first flap reconstructions of tongue cancer employed the pectoralis major myocutaneous flap (PMMF). Due to the absence of taste buds in this flap, the taste score was the lowest domain in advanced OTSCC treated with PMMF reconstruction, regardless of the extent of glossectomy (partial vs. subtotal). Currently, PMMF is rarely used due to the microsurgical technique improvements; however, it can be employed as a soft tissue component to a large reconstruction, regardless of the patient’s general status, without needing a microvascular anastomosis [33]. Free flap reconstruction has become the better technique to repair medium or large defects after H&N cancer resection. This technique has many advantages, such as the selection of the most adapted tissue for the reconstruction, improved cosmetic and functional outcomes, and 3-dimensional freedom of the flap positioning. Compared to no free-flap reconstruction, a better taste score was shown in free-flap repairing OSCC [31]. Regarding the type of free flap, no difference was found between anterolateral thigh perforator free flaps (ALTFF) and radial free forearm flaps (RFFF) in OTSCC patients. All patients experienced taste impairments, regardless of the surgical reconstruction and the follow-up period (6–12 months). Similarly, comparing PMMF and RFFF reconstruction in OTSCC patients, Li W. et al. did not show any significant differences in the taste domain [8]. Additionally, no significant differences were found between OSCC patients treated with or without microvascular free flaps [26]. Recently, Lu et al. proposed a modified anterior–posterior tongue rotation flap for pT2-OTSCC, demonstrating no patients experienced taste impairment at 12 and 24 months [24]. Interestingly, Elfring et al. compared different types of lingual nerve surgical procedures in OPSCC patients. Despite the reconstruction, the affected tongue side had significantly reduced taste sensitivity compared to the unaffected side. Compared to controls, patients exhibited significantly poorer taste outcomes of the affected site, regardless of the surgical technique (reanastomosed, cable-grafted and lingual nerve cut), suggesting that lingual nerve repair had limited success in restoring gustatory function [35].

The severity of dysgeusia depends on the extent of surgical resection. Preserving more than the 50% of the tongue base, taste acuity and its detection threshold are improved. Additionally, reconstruction modalities influence taste perception. Free flaps, regardless of the type, significantly improve dysgeusia compared to no-free flaps, as shown in Table 1.

## 3. Dysgeusia Detection Methods

Dysgeusia is assessed clinically by measuring the detection and/or recognition threshold values of five basic tastes: sweet, bitter, sour, salty, and umami. Umami, sweet, and bitter are detected by G protein-coupled receptors, while salty and sour are detected via membrane channel proteins. Oral chemosensory responses also include trigeminal stimuli for detecting spicy and cooling sensations via C-fiber signaling. Diagnosis of dysgeusia can be obtained by objective and subjective methods. The objective methods used are chemical gustometry [6,10,13,18,35] and electrogustometry (EGM). The first can be performed through paper strips or aqueous solutions. The substances are offered to the patients in different concentrations to allow the qualitative and quantitative analysis of the flavors, evaluating the type of sensitivity loss and its intensity. The substances commonly used are sucrose for the perception of sweet taste, sodium chloride for salty, citric acid for sour, and quinine sulphate or caffeine for bitter. For the umami flavor, the combination of monosodium glutamate and inosine monophosphate-3 is commonly used. EGM represents the electrical stimulation of the gustatory receptors. It is not a qualitative test, because it defines the electric density necessary to produce a gustatory perception. The subjective analysis of dysgeusia can be performed by questionnaires where patients report any daily taste changes. The severity of the taste dysfunction can be graded using several intensity scales [11,23,25,30]. Moreover, there are several H&N disease-specific QOL questionnaires, such as the University of Washington QOL Questionnaire (UW-QOL), Functional Assessment of Cancer Therapy-H&N (FACT-H&N), the European Organization for Research and Treatment of Cancer QOL Questionnaire-H&N module (EORTC-H&N35) and 14-item Oral Health Impact Profile (OHIP-14). Their number reflects the absence of a ‘gold standard’ module; however, many researchers used the UW-QOL and/or EORTC-H&N35 questionnaires [7,8,17,19,21,22,24,27,28,29,31,33,34,36] since they are extensively validated in the OSCC patients. According to Tomita et al., a partial glossectomy critically lowers taste sensation in the remaining tongue. Indeed, the patient’s taste threshold was significantly higher than the controls. However, no significant difference in taste detection threshold was found between the remaining tongue and the posterior wall of the oropharynx, comparing OTSCC patients treated by partial or subtotal glossectomy [6]. Recently, a higher increase in the dislike of spicy and salty tastes has been reported, both during treatment and follow-up, in OSCC patients. Only the sweet taste seems to stimulate strong and pleasant perceptions. Bitter, umami, and fat tastes were the most altered, especially during the post-treatment period [18].

The frequency of bitter taste hypogeusia was significantly higher compared with the other tastes. Especially da Chuna et al. showed a bitter taste alteration in 80% of OSCC patients, while sweet, salty, and sour hypogeusia were detected in 20–30% of them. It can be related to the impairment of the gustatory papillae in the base of the tongue, common in oropharyngeal tumors, while the lower change of sour stimulus can be attributed to a biological mechanism to warn and protect us associated with the rejection of certain foods [13]. Finally, Pingili et al. significantly correlated the sensory difficulty for a taste sensation with malnutrition in patients affected by OSCC and OPSCC treated by surgery and/or radiation therapy and/or chemotherapy. No significant differences regarding both the follow-up time (3 months vs. 6 months) and the treatment type (surgery alone, surgery and radiation therapy, surgery, radiation therapy and chemotherapy) emerged [22].

## 4. Management of Dysgeusia

Taste alteration varies with each patient and specific suggestions for dysgeusia management must be personalized. Firstly, physicians should focus on preventing treatment-related symptoms, offering additional support during therapy. Thus, regular reporting of symptoms by patients also helps the physician to provide adapted therapeutic modalities. Self-care strategies should be adopted daily by the patient, such as consuming cold or lukewarm foods and frozen fruits, adding more seasoning and/or sugar to foods, choosing protein products with a mild flavor, reducing consumption of bitter or metallic tasting foods, drinking more water, eating smaller meals several times a day and avoiding foods with strong smells. Furthermore, optimal oral hygiene and tongue brushing are fundamental for taste acuity improvements. Zinc supplementation is commonly used to treat taste alterations. Indeed, it is an essential mineral for the development, maintenance, and proliferation of taste buds, as well as for the regulation and repair of taste function. Recently, Khan et al. demonstrated significantly higher recognition taste thresholds of sweet and sour in patients taking zinc supplements. However, this treatment should be used with caution as excessive zinc intake can negatively impact the immune system [10]. Other treatment methods could be alpha-lipoic acid, glutamine, biotin, or lactoferrin supplementation, Ginkgo biloba use, and saliva substitutes or stimulants.

Oral supplementation should be included in a wider assessment of an adequate nutrition plan. Indeed, in cancer patients, particularly in those who suffer from taste disorders, the nutritional condition should be evaluated and managed quickly in a targeted way in each patient, based on nutritional conditions, clinical status, expected treatments and expected outcomes [38]. Personalized nutritional counseling and the drafting of an appropriate nutritional plan, based on spontaneous and tolerated food intake and its effectiveness, are an integral part and the first steps of adequate nutritional therapy. A relational activity aimed at empowering the subject and overcoming the “descriptive” dietary approach can positively influence prognosis in the patient and generally improve the quality of life [38]. In particular, if the calorie-protein intake is less than 75% of the required amount, the patient is selected for counseling and the development of a personalized diet plan is recommended, prepared by competent staff such as a dietician, in agreement with the patient, and re-evaluated according to various individual needs [39].

Additionally, pharmacological, surgical, or physical treatments have been proposed, such as tricyclic antidepressants and benzodiazepines, theophylline, and proton-pump inhibitors; magnetic stimulation, lingual nerve surgery; local anesthesia, cryotherapy, acupuncture, and photobiomodulation [37]. However, further research should be performed to confirm the efficacy of these therapeutic strategies in the treatment of dysgeusia.

## 5. Conclusions

Survival remains the primary endpoint of oncological patients; however, the importance of QOL has been recognized over the last decade. Indeed, taste changes have been reported to reduce treatment compliance, impair the immune system, alter food intake, and cause social and emotional distress. It is impossible to generalize results reported in the literature to all oral and oropharyngeal cancer patients due to several limitations. The studies conducted on this topic are characterized by small samples size, different pathological stages (mainly advanced OSCC and/or OPSCC), heterogeneous therapeutic protocols and a strong prevalence of male-gendered cohorts, while only few studies included a matched control group. Furthermore, taste alterations are frequently detected by subjective self-reporting methods using different severity scales and types of questionnaires. Therefore, further prospective studies using standardized protocols and tools, and a greater sample size are needed to validate the results.

## Figures and Tables

**Table 1:** Data collection of literature study, regarding therapy induced dysgeusia in OSCC and OPSCC included in the review.

Authors (Year) Country [Ref]	Study Design	Tumor Site	Pathological Stage	Total Cases	Treatment	Taste Assessment	Data Collection (m) *	Results
Pingulii et al., (2021) India [22]	Prospective	Oral cavity Oropharynx	All 48 I/II 49 III/IV	97	RS, RS + RT, SR + RT + CMT	QOL-H&N35	3–6	Taste sensation impairment associated with patient malnutrition.
Da Cunha et al., (2020) Brazil [13]	Cross sectional	Oral cavity Oropharynx	All 6 I/II 25 III/IV	31	RT + CMT	Chemical Gustometry	0	pT is significantly associated with hypogeusia. Bitter disorder is significantly higher compared other tastes.
Vempati et al., (2020) USA [23]	Prospective	Oral cavity	All	34	CMT + RT + SRS	NCI CTCAE (v. 4.0)	0–24	High prevalence (90%) of acute (<90 days) and tardive (>90 days) dysgeusia after SRS.
Epstein et al., (2020) USA [18]	Prospective	Oropharynx	All	10	IMRT +/- CMT	NCI CTCAE (v. 4.0) Likert Scale STTA	0–24	Sweet stimulates a pleasure taste perception. Spicy and bitter tastes stimulate the most dislike perception. Prevalence and severity of dysgeusia are significantly higher in smokers compared to non-smokers.
Lu et al., (2020) China [24]	Cross-sectional	Tongue	II	21	RS + Flap	UW-QOL (v.4)	12–24	No patient complains dysgeusia, regardless the follow-up period.
Palmieri et al., (2019) Brazil [11]	Prospective	Oral cavity	All 2 I/II 18 III/IV	20	RT + CMT	NCI CTCAE (v. 4.0)	Weekly during RT (1 to 6 w)	Severe dysgeusia is experienced from the 3° week of RT, with a peak during the 5° week of RT.
Khan et al., (2019) Pakistan [10]	Case Control	Oral cavity	All	68	RT + CMT	Chemical gustometry	0–7/8–11/12 w	Salty and sour postoperative recognition thresholds are significantly better in cases compared to controls. Sweet is the most altered taste.
Abbas et al., (2019) Pakistan [21]	Cross sectional	Oral cavity	All	59	RS +/- RT +/- CMT	UW-QOL (v.4)	0–12	Advanced tumors have significantly worse taste score compared to early tumors. Advanced OTSCCs have significantly worse taste score compared to early cheek tumors.
Moroney et al., (2018) Australia [25]	Prospective	Oropharynx	All 1 II 3 III 72 IV	76	IMRT + CMT	NCI CTCAE (v. 4.0) FOIS	Weekly during IMRT (1 to 7 w) 2–4–12 w post-IMRT	The prevalence and severity of dysgeusia increase during the therapy and 2–postoperative weeks, with a sharped increase from the 3° and 4° week of treatment. 12–weeks post therapy, the dysgeusia is experienced by 80% of patients.
Yue et al., (2018) China [26]	Case control	Oral cavity	All	139	RS +/- Free Flap	UW-QOL (v.4)	12	No significant difference of taste score between cases and controls. Taste represented the worst domain.
Galitis et al., (2017) Greece [19]	Prospective	Oral cavity	All	10	RS +/- RT/CMT	UW-QOL (v.4) QOL-H&N35	Pre RT- End of RT- 3m post RT	Pre-RT dysgeusia: 40% End-RT dysgeusia: 88% 3 months follow-up dysgeusia: 50% 3-fold increased symptoms during RT.
Naqvi et al., (2017) Pakistan [27]	Prospective	Oral cavity Oropharynx	All 22 I/II 12 III/IV	34	RS +/- RT/CMT	UW-QOL (v.4)	0–6	No significant difference of taste score between baseline and follow-up (6 months).
Yan et al., (2017) China [28]	Prospective	Oral cavity	III/IV	55	RS +/- RT	UW-QOL (v.4)	0–3–12 96	Significant difference of taste score between survival and nonsurvival patients, 12–and 96–months post therapy. Survival patients showed a significant taste score difference between baseline and long-term (8 years) follow-up.
Li W. et al., (2016) China [8]	Prospective	Tongue FOM	All	41	RS + Flap +/- CMRT	UW-QOL (v.4) OHIP 14	0–3	No significant difference regarding the type of flap reconstruction. Taste is one of the worse domains.
Yuan et al., (2016) China [29]	Prospective	Tongue	All	67	RS + Flap	UW-QOL (v.4) OHIP 14 QOL-H&N35	0–6–12	Taste score significantly worsened 6–and 12–months post-therapy, compared to baseline. No significant difference regarding the type of flap. Taste score represents the worst domain.
Hayashi et al., (2016) Japan [30]	Prospective	Oral cavity	All	46	PBT+ CMT	NCI-CTCAE (v. 4.0)	0–12	Dysgeusia: 64% Moderate dysgeusia: 57% Severe dysgeusia: 0%
Singh et al., (2016) India [9]	Case Control	Oral cavity	III	24	RT	RTOG 0435 H&N (CTCAE)	0–1–3	No significant difference of severity of dysgeusia between cases and controls, regardless follow-up period. Cerrobend stent does not contributed to improvement of taste alteration
Sapir et al., (2016) USA [7]	Prospective	Oropharynx	III/IV	73	IMRT	UW-QOL (v.4) QOL-H&N 35 XQ	0–1–3–6–12	Severity of dysgeusia is correlated to mean radiation dose. This dose tends to decrease during follow-up, Severe dysgeusia is significantly correlated with xerostomia. Taste score significantly worsened 1month post-therapy
Chen et al., (2015) Taiwan [20]	Prospective	Oral cavity	All 8 I/II 69 III/IV	77	RT	MMA-MSS-moo	0–4–8	Taste alterations represent the main symptom correlated to radiation-induced oral mucositis. Taste score significantly worsened 4- and 8-months post-therapy.
Fang et al., (2014) China [31]	Prospective	Oral cavity	III/IV	49	RS + Flap + ND	UW-QOL (v.4)	12	Free-flap reconstruction significantly improves the taste function compared to no-free flap reconstruction.
Agarwal et al., (2014) India [32]	Prospective	Tongue	All	39	RS + ND +RT	UW-QOL (v.4)	0–12	Taste score is significantly worst 12-months post-therapy compared to baseline.
Tomita et al., (2014) Japan [6]	Case Control	Tongue	All	39	CMT + RS +Flap + ND	Chemical Gustometry	0	Dysgeusia: 50% Taste threshold of cases treated by partial glossectomy is significant lower respect to controls. Conservation of > 50% of tongue significantly improves the taste function.
Fang et al., (2013) China [33]	Prospective	Tongue FOM	All 5 I/II 16 III/IV	21	RS + Flap	UW-QOL (v.4)	12	Taste score is one of the worst domains. No significant different between partial and subtotal glossectomy.
Zhang et al., (2013) China [34]	Case control	Tongue	All 48 I/II 15 III/IV	63	RS	UW-QOL (v.4)	12	Patients < 40 years have a significantly worse taste score compared to patients > 40 years. Taste represents the worst domain.
Oskam et al., (2013) Netherlands [17]	Prospective	Oral cavity Oropharynx	III/IV	80 27	RS + Flap + RT	QOL-H&N35	0–6–12 96–132	Long-term survival patients show significant better taste score at baseline and 1-year post-therapy, compared to nonsurvival patients.
Elfring et al., (2012) Canada [35]	Case Control	Oropharynx	All	60	RS + Flap +/- RT or CMT	Chemical Gustometry	0	Affected side of cases have significant worse taste functions compare to controls and to unaffected patients’ side.
Airoldi et al., (2011) Italy [36]	Cross-sectional	Oral cavity	All 8 I/II 28 III/IV	36	RS + Flap + RT	QOL-H&N 35 Dische scale	0	Dysgeusia: 33% Ageusia: 31%

w: week; m: month; FOM: floor of mouth; RT: radiation therapy; CMT: chemotherapy; SRS: stereotactic radiosurgery; IMRT: intensity modulated radiation therapy; RS: surgical resection; CMRT: chemoradiotherapy; PBT: proton beam therapy; ND: neck dissection; v.: version; NCI: National Cancer Institute; CTCAE: Common Terminology Criteria for Adverse Events; STTA: Scale of Subjective Total Taste Acuity; UW-QOL: University of Washington Quality of Life Questionnaire; FOIS: Functional Oral Intake Scale; QOL-H&N35: Quality of Life-Head & Neck 35; OHIP 14:14-item Oral Health Impact Profile; RTOG: Radiation Therapy Oncology Group; XQ: xerostomia questionnaire; MMA-MSS-moo: MacDibbs Mouth Assessment-MacDibbs Symptom Score-modified for oral cavity cancer; OTSCC: oral squamous cell carcinoma. * unless otherwise specified.
